# How to Prevent Injuries in Alpine Ski Racing: What Do We Know and Where Do We Go from Here?

**DOI:** 10.1007/s40279-016-0601-2

**Published:** 2016-08-01

**Authors:** Jörg Spörri, Josef Kröll, Matthias Gilgien, Erich Müller

**Affiliations:** 1 Department of Sport Science and Kinesiology, University of Salzburg, Schlossallee 49, 5400 Hallein-Rif, Austria; 2 Department of Physical Performance, Norwegian School of Sport Sciences, PO Box 4014, Ullevål Stadion, 0806 Oslo, Norway; 3 Center of Alpine Sports Biomechanics, St. Moritz Health and Innovation Foundation, Via Somplaz 1, 7500 St. Moritz, Switzerland

## Abstract

Alpine ski racing is known to be a sport with a high risk of injury and a high proportion of time-loss injuries. In recent years, substantial research efforts with regard to injury epidemiology, injury etiology, potential prevention measures, and measures’ evaluation have been undertaken. Therefore, the aims of this review of the literature were (i) to provide a comprehensive overview of what is known about the aforementioned four steps of injury prevention research in the context of alpine ski racing; and (ii) to derive potential perspectives for future research. In total, 38 injury risk factors were previously reported in literature; however, a direct relation to injury risk was proven for only five factors: insufficient core strength/core strength imbalance, sex (depending on type of injury), high skill level, unfavorable genetic predisposition, and the combination of highly shaped, short and wide skis. Moreover, only one prevention measure (i.e. the combination of less-shaped and longer skis with reduced profile width) has demonstrated a positive impact on injury risk. Thus, current knowledge deficits are mainly related to verifying the evidence of widely discussed injury risk factors and assessing the effectiveness of reasonable prevention ideas. Nevertheless, the existing knowledge should be proactively communicated and systematically implemented by sport federations and sport practitioners.

## Key Points

**Table:** 

In the context of alpine ski racing to date, various potential injury risk factors and prevention measures have been suggested in the literature. However, statistical evidence has been proven for only a few of them, and only one prevention measure has been demonstrated to significantly reduce injury risk.
Future research should aim to fill the lack of knowledge revealed by this review of the literature. In principle, our current knowledge is limited within all four steps of van Mechelen’s ‘sequence of prevention’ model. Major deficits were observed to be (i) the assessment of evidence of potential injury risk factors; and (ii) the evaluation of effectiveness of etiology-derived injury prevention measures.
An absent, yet important perspective is that of monitoring and preventing injuries at the youth level. In this field, additional research efforts would be desirable.

## The Framework of Injury Prevention

**Fig. 1:**
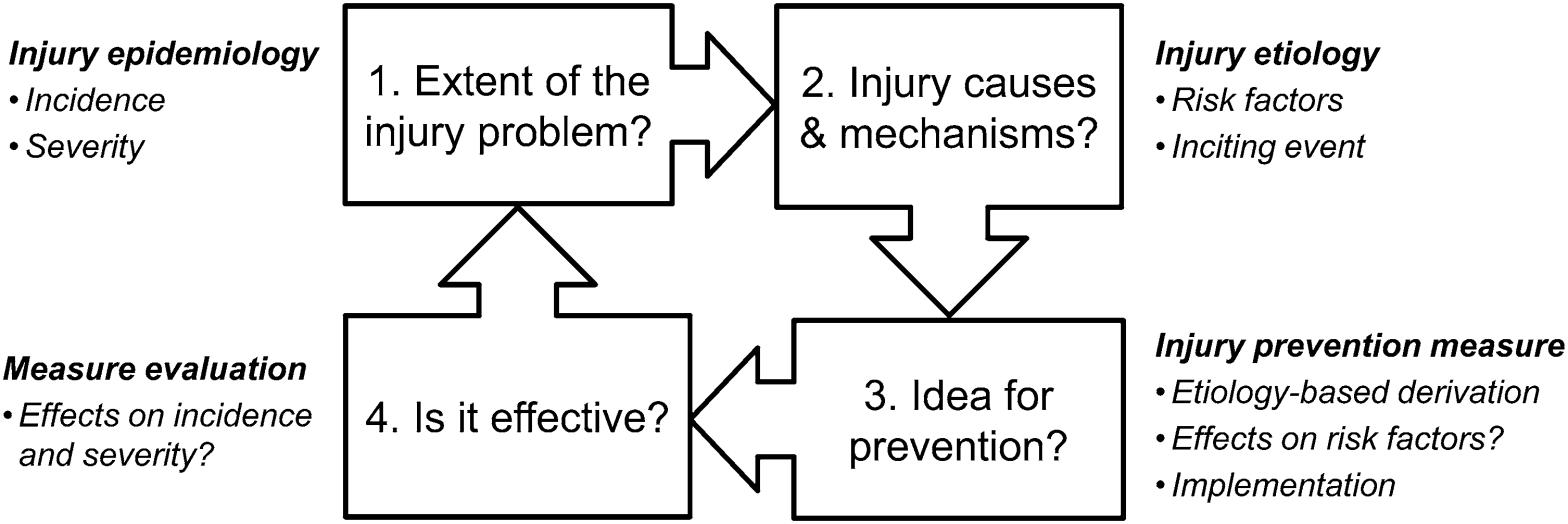
Four-step sequence of injury prevention research. (adapted from van Mechelen et al. [1], with permission)

The development and implementation of effective prevention measures are essential actions for protecting athletes’ health. In this context, several conceptual models have provided a methodological framework for the systematic derivation and assessment of injury prevention strategies [1–4]. One common framework for approaching injury prevention research can be found in van Mechelen’s ‘sequence of prevention’ model (presented in Fig. 1), and a multifactorial model of injury causation [1, 2, 4]: (i) injury epidemiology should be described by reporting the injury incidence and severity; (ii) injury etiology should be established by investigating the risk factors and describing the injury situations/mechanisms; (iii) prevention measures should be derived from etiological knowledge and should subsequently be implemented; (iv) finally, the prevention measures implemented should be evaluated by repeating step one. In an organizational setting (such as that of international sports federations), in addition to the aforementioned four-step sequence, the task of ‘risk communication’ should receive special attention because without a sophisticated communication strategy, effective prevention measures and higher-level risk mitigation strategies will not be accessible to stakeholders [3].

In recent years, the aforementioned conceptual models have been major pillars of the risk management process within leading sports governing bodies, such as the International Football Association (FIFA) or the International Ski Federation (FIS) [5, 6]. Both FIFA and the FIS have recognized their key responsibilities to protect their athletes’ health and have systematically implemented research-based injury surveillance and risk mitigation programs. Due to the high-risk nature of alpine ski racing (occurrence of high kinetic energy/forces along with an error-prone human–environment interaction), skier safety is a priority for the FIS [7]. Accelerated by the apparent injury prevention purpose of the FIS, substantial interdisciplinary research efforts have been undertaken in recent years.

Therefore, the aims of this review of the literature were twofold: (i) to provide a comprehensive overview of what is known about injury prevention in alpine ski racing; and (ii) to derive potential perspectives for future research.

## Methodological Aspects

This is a comprehensive review of what is known about injury prevention in alpine ski racing. Given the current stage of knowledge in this area, a narrative (non-systematic) review was considered to be methodologically more appropriate than a systematic review because most of the existing knowledge is based on expert perceptions and/or descriptive accounts of injury risk associations, with only a very small number of studies contributing higher level evidence. Consequently, this article primarily provides an overview of exploratory research (a frequent aim of a narrative review) rather than a collation of empirical evidence to answer a specific research question (the inherent aim of a systematic review).

**Fig. 2:**
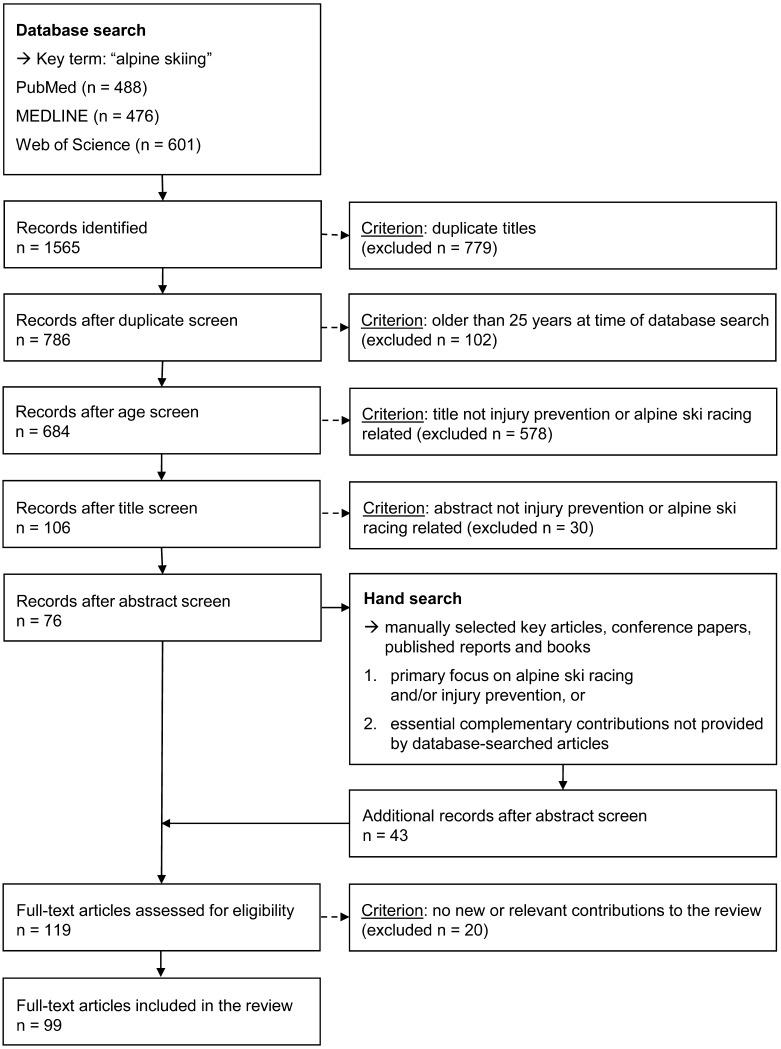
Search strategy, exclusion criteria, and article selection process

Relevant studies were identified by searching three databases (PubMed, MEDLINE, and Web of Science—accessed 31 January 2016). The key search term used was ‘alpine skiing’ and the major focus was on injury-related articles in the context of alpine ski racing. A flow diagram describing the detailed search strategy, exclusion criteria, and article selection process is shown in Fig. 2. An overview of the articles selected for this review (categorized according to their assignment to the four steps of van Mechelen’s ‘sequence of prevention’ model) is presented in Table 1.

**Table 1:** Articles included in the review (categorized according to their assignment to the four steps of van Mechelen’s ‘sequence of prevention’ model)

	Step 1: Injury epidemiology	Step 2: Injury etiology	Step 3: Injury prevention measure	Step 4: Measure evaluation
Articles included based on database search (focus: alpine ski racing)	[8–22, 24]	[9, 10, 15–17, 20–22, 24–28, 30, 31, 33, 34, 36, 38, 40–42, 47–49, 51–53]	[6, 7, 15, 17, 19, 24–28, 34, 36, 48, 49, 51–53, 57, 59–61, 65–67, 73, 76, 77, 79, 81, 84, 90–92, 94, 95, 98]	[47]
Manually selected key articles, conference papers, book sections, books, or published reports	[23]	[29, 32, 35, 37, 39, 43–46, 50, 54–56]	[35, 37, 43, 44, 46, 50, 58, 62–64, 68–72, 74, 75, 78, 80, 82, 83, 85–88, 93, 96, 97, 99]	

## What is Known About Injury Prevention in Alpine Ski Racing?

### Injury Epidemiology

#### Injury Incidence

In contrast to injury rates in recreational alpine skiing that have been documented since the early 1970s, epidemiological studies assessing alpine ski racing are limited. Only two single-event studies and two cross-sectional studies attributable to the time span before the winter season of 2006/2007, and that were not older than 25 years, were identified [8–11]. As a result of this lack of data, in 2006 the FIS established an Injury Surveillance System (ISS) that records injuries among world cup (WC) athletes based on retrospective interviews [12].

Among the Olympic winter sports, alpine ski racing is known to be a sport with an above average risk of injury [13, 14]. Recent studies from the FIS ISS reported absolute injury rates of 36.7 and 36.2 injuries per 100 WC athletes per season [15, 16]. Injury incidence was found to increase from slalom (4.9 injuries/1000 runs) to giant slalom (9.2 injuries/1000 runs) to super-G (11.0 injuries/1000 runs) to downhill (17.2 injuries/1000 runs) [15]. However, when the number of injuries was considered in relation to effective exposure time (i.e. per hour of skiing), all disciplines were found to be equally dangerous on the WC level [17]. As many as 45 % of all injuries in WC alpine ski racing were found to occur during official competitions or world championships, and only 25.1 % during regular team training on snow [15, 18]. The most frequently injured body parts were found to be the knee (35.6 %) and the lower leg (11.5 %), with a rupture of the anterior cruciate ligament (ACL) being the most frequent diagnosis (13.6 % of all injuries) [15, 18]. Other frequently injured body parts were the lower back, pelvis, sacrum (11.5 %), the hand, finger and thumb (8.9 %), as well as the shoulder (6.8 %) [15]. Head/face injuries accounted for 8.4 % of all injuries [15], whereas 3.5 head/face injuries per 100 WC athletes per season were found to occur [19].

Studies including cohorts from national ski associations (not limited to athletes at the WC level) reported comparably high injury rates; however, due to differences in reporting methods, no direct comparison is feasible [20–24]. Similar to studies at WC level, the most frequent injured body parts were found to be knee or lower-leg injuries [21–23]. One of these studies found a higher risk for traumatic injuries during the winter season, while during the summer season a higher risk for overuse injuries was reported [23].

#### Injury Severity

During winter seasons of 2006/2007 and 2007/2008, 81.2 % of all injuries in WC alpine ski racing were time-loss injuries that resulted in an absence in training and/or competition [15, 18]. Nearly one-third (30.8 %) of all injuries were reported to be severe (>28 days of absence) [15, 18]. Throughout six consecutive winter seasons (2006/2007–2011/2012), these initial values were confirmed (time-loss: 80.9 %; severe: 35.6 %) [16]. With regard to the most frequently injured body parts, it was found that 54.4 % of all knee injuries and 31.8 % of all lower-leg injuries were severe [15]. Similar results were found for junior athletes [21]. Additionally, severe traumatic head injuries were reported to account for 23.7 % of all head injuries in WC alpine ski racing [19].

### Injury Etiology

Prior to being able to develop effective preventative measures that reduce the risk of injury, injury causes need to be well understood [1]. In this context, it has been suggested that prevention measures should be derived from risk factors [1]. Following this approach, Tables 2, 3, 4 and 5 (left-hand side) present an overview of the risk factors reported in the literature to date. Within the subsequent sections, these factors will be described in more detail.

**Table 2:** Athlete-related injury risk factors and potential injury prevention measures in alpine ski racing (ordered according to their scientific status and alphabetically)

Athlete-related injury risk factor	Status	Potential athlete-related injury prevention measure	Status
Adverse crash behavior [34, 35]	P	Awareness training for injury mechanisms [35, 57]; specific conditioning training [35]	1
Fatigue (due to schedule/jetlag) [34, 35]	P	Appropriate schedule [35]; systematic stress monitoring;^a^ superior fitness [35]	1, (3)
Insufficient adaptability [34, 35]	P	Explicit training of ‘adapting skills’ (injury prevention purpose)^a^	1
Low peripheral body temperature [34, 35]	P	No competitions below −27 °C [36]; regimentation of thicker racing suits [35, 37]	1, (3)
Poor individual responsibility/risk management [34, 35]	P	Awareness campaigns/athletes’ education programs^a^	1
Poor mental skills [34, 35]	P	Explicit training of ‘mental skills’ (injury prevention purpose)^a^	1
Pre-injury [34, 35]	P	Mandatory fulfillment of return to sport criteria (verified by screening methods) [58–60]	1
Unfavorable anthropometrics [34, 35]	P	NA	NA
Fatigue (within a course or training session) [25, 34, 35]	A, P	Superior fitness [25, 34, 35]; shorter race tracks;^a^ active on-hill recovery [66]	1, (3)
Inappropriate tactical choices [26, 34, 35]	A, P	Explicit training of adequate ‘tactical decisions’ (injury prevention purpose)^a^	1
Insufficient physical fitness [25, 34, 35]	A, P	Awareness campaigns [35]; mandatory score limits for physical fitness tests [35]	1
Technical mistakes [26, 34, 35]	A, P	Training of a stable ‘skiing technique’;^a^ specific balance/neuromuscular training [27, 67]	1, (3)
Female/male sex [10, 15, 16, 22, 24]	E^b^	NA	NA
Insufficient core strength/core strength imbalance [24]	E	Awareness campaigns/athletes’ education programs [24]	1, (3)
High skill level [20]	E	NA	NA
Unfavorable genetic predisposition [34, 35, 40]	E, P	NA	NA

Numbers in brackets indicate partially completed steps

*P* ‘expert stakeholder perception’ (i.e. theory- and practical experience-based expert belief), *A* ‘indirect association with injury risk’ (i.e. findings by systematic video analyses, as well as biomechanical field or simulation studies that report association between an injury risk factor and individual injury cases or injury-related variables), *E* ‘statistical evidence’ (i.e. significant relations between an injury risk factor and injury rates determined by epidemiological studies), *1* basic idea for etiology-derived prevention measures, *3* implemented prevention measures, *NA* not applicable

^a^Authors’ suggestion only

^b^Existence of contradicting results in the literature

**Table 3:** Equipment-related injury risk factors and potential injury prevention measures in alpine ski racing (ordered according to thematic key areas, their scientific status, and alphabetically)

Equipment-related injury risk factor	Status	Potential equipment-related injury prevention measure	Status
Ski-plate-binding-boot system			
Heavy equipment weight [34, 35]	P	Lighter equipment components [34, 35]	1
High standing height due to the ski-plate-binding-boot unit [34, 35]	P	Reduced standing height [34, 35, 43, 71]	1, 2, 3
Skis with high torsional stiffness/homogenous bending lines [34, 35]	P	Skis with reduced torsional stiffness [34, 35, 72] or in-homogenous bending line [34, 35]	1, (2)
Stiff ski boots [34, 35]	P	Less-stiff boots [34, 35, 73]; correct boot settings [74]	1, (3)
Highly-shaped skis [28, 34, 35, 47]	E, A, P	Less-shaped skis [6, 28, 34, 35, 44, 47, 51, 76–78]	1, 2, 3, 4
Short skis [34, 35, 47]	E, P	Longer skis [6, 34, 35, 47, 71, 76, 78]	1, 2, 3, 4
Wide skis [34, 35, 47]	E, P	Skis with reduced profile width [6, 34, 35, 47, 71, 76, 78, 81]	1, 2, 3, 4
Gates			
Gates with high resistance [25, 34, 35]	A, P	Alternative panels/poles with less resistance or optimized release mechanism [25, 35]	1, 3
Bindings			
Non-release/inadvertent release of bindings [27, 34, 35]	A, P	Development of new, more sophisticated binding/binding-plate concepts [65, 82, 83]	1
Protective devices			
Insufficient body protection [34, 35]	P	Body protectors, knee orthoses and airbag systems [84, 85]	1, 3
Insufficient head protection [25, 34, 35, 48]	A, P	More sophisticated helmet standards [48]	1, 3

Numbers in brackets indicate partially completed steps

*P* ‘expert stakeholder perception’ (i.e. theory- and practical experience-based expert belief), *A* ‘indirect association with injury risk’ (i.e. findings by systematic video analyses, as well as biomechanical field or simulation studies that report association between an injury risk factor and individual injury cases or injury-related variables), *E* ‘statistical evidence’ (i.e. significant relations between an injury risk factor and injury rates determined by epidemiological studies), *1* basic idea for etiology-derived prevention measures, *2* prevention measures with significant effects on injury-related variables (i.e. injury risk factors), *3* implemented prevention measures, *4* prevention measures with an evaluated, significant effect on injury risk

**Table 4:** Course-related injury risk factors and potential injury prevention measures in alpine ski racing (ordered according to their scientific status and alphabetically)

Course-related injury risk factor	Status	Potential course-related injury prevention measure	Status
High skiing speed combined with terrain transitions [34, 35]	P	Course settings that radically slow down skiers before key sections [34, 94]	1, (3)
High skiing speed combined with small turn radii [17, 34, 35]	A, P	Increased gate offset, shorter linear gate distance [49, 94]	1, 2, 3
High skiing speed in general [15, 17, 34, 35]	A, P	Course setting/equipment interventions/steeper terrain [35, 49, 71, 77, 93–95]	1, 2, (3)
Inappropriate jump construction [17, 25, 27, 34, 35, 52, 53]	A, P	Decreased take-off speeds/angles, steep landings [35, 53]; trunk control training [52]	1, 2, (3)
Inappropriate net positions [25, 34, 35]	A, P	Finding optimal net positions by simulations [96]; no B-nets in front of A-nets [35]	1, (3)
Limited spill zones [25, 34, 35]	A, P	Larger spill zones (if necessary by constructional adaptations) [35]	1, (3)
Poor visibility [26, 34, 35]	A, P	Repeated blue coloration to enhance the contrasts on the snow surface [26, 35]	1, 3

Numbers in brackets indicate partially completed steps

*P* ‘expert stakeholder perception’ (i.e. theory- and practical experience-based expert belief), *A* ‘indirect association with injury risk’ (i.e. findings by systematic video analyses, as well as biomechanical field or simulation studies that report association between an injury risk factor and individual injury cases or injury-related variables), *1* basic idea for etiology-derived prevention measures, *2* prevention measures with significant effects on injury-related variables (i.e. injury risk factors), *3* implemented prevention measures

**Table 5:** Snow-related injury risk factors and potential injury prevention measures in alpine ski racing (ordered according to their scientific status and alphabetically)

Snow-related injury risk factor	Status	Potential snow-related injury prevention measure	Status
Aggressive snow conditions [26, 34, 35]	A, P	Additional water preparation [34, 35]; adequate equipment setups^a^	1, (3)
Changing snow conditions [26, 34, 35]	A, P	Avoidance of alterations in snow preparation techniques [26, 34, 35]	1, (3)
Too bumpy/smooth snow surface [26, 34, 35]	A, P^b^	NA	NA
Water-injected/non-injected snow [26, 34, 35]	A, P^b^	NA	NA

Numbers in brackets indicate partially completed steps

*P* ‘expert stakeholder perception’ (i.e. theory- and practical experience-based expert belief), *A* ‘indirect association with injury risk’ (i.e. findings by systematic video analyses, as well as biomechanical field or simulation studies that report association between an injury risk factor and individual injury cases or injury-related variables), *1* basic idea for etiology-derived prevention measures, *3* implemented prevention measures, *NA* not applicable

^a^Authors’ suggestion only

^b^Existence of contradicting results in the literature

As stated by van Mechelen et al. [1], to merely establish risk factors might not be enough; the inciting events (i.e. the events leading to injury situations and injury mechanisms) must also be identified [1, 4]. For alpine ski racing, it is known that nearly all injuries occur while the skier is turning (80 %) or landing (19 %) [25]. Injuries to the head and upper body mainly resulted from crashes (96 %), while the majority of knee injuries (83 %) occurred while the skier was still skiing [25]. With regard to head injuries, it was found that the main impact was most often caused by forceful contact with the snow surface, while collisions with safety nets/materials and gates were less frequent [26].

With regard to the ACL, three main alpine ski-racing-specific injury mechanisms were identified as the so-called ‘slip-catch’, ‘dynamic snowplow’, and ‘landing back-weighted’ [27]. The slip-catch mechanism accounts for approximately half of the ACL injuries, and typically occurs while turning (mainly while steering out of the fall line) [27]. The skier becomes out of balance in the backward and inward direction, and loses snow contact and pressure on the outer ski [27]. Subsequently, the inside edge of the outer ski abruptly catches the snow surface, leading to excessive knee joint compression, knee valgus, and internal rotation [28]. A similar order of events and similar loading patterns were ascribed to the dynamic snow-plow mechanism; however, in this mechanism, it is the inside edge of the inner ski (not the outer ski) that abruptly catches the snow surface [27]. The landing back-weighted mechanism typically occurs during jump landings. During the flight phase, the skier loses balance in the backward direction due to a backward-directed angular momentum obtained at the jump take off [27]. As a result, the skier lands on the ski tails with a large clap angle [27]. At initial contact with the ground, a forward directed angular momentum rotates the skis forward while the skier falls backward, resulting in tibiofemoral compression and a boot-induced anterior drawer of the tibia relative to the femur [27]. Within this period of initial contact, internal tibia rotation might also play an important role [29]; however, there is also existing evidence that indicates that during the period of the initial ground contact, only small forces are transmitted to the ACL, and that the ACL rupture may occur later while recovering from the back-seated position after a failed landing [30–33]. During this period, a combination of highly loaded quadriceps muscles and anteriorly-directed ground reaction forces, which result from a strong deformation of the ski tails when landing back-weighted, might increase ACL loading [31].

#### Athlete-Related Injury Risk Factors

Athlete-related risk factors that were reported based on expert stakeholder perceptions were athletes’ ‘adverse crash behavior’, ‘fatigue due to schedule/jet lag’, ‘insufficient adaptability’, ‘low peripheral body temperature’, ‘poor individual responsibility/risk management’, ‘poor mental skills’, ‘pre-injury’, and ‘unfavorable anthropometrics’ [34, 35]. With regard to ‘low peripheral body temperature’, it is known that cold conditions facilitate body heat transfer to the environment, potentially leading to hypothermia and frostbite [36, 37]. With regard to ‘poor individual responsibility/risk management’, it has been illustrated that athletes sometimes gamble with their health rather than miss an important competition or risk their place on the team [38]. In addition, with regard to ‘pre-injury’, 72 % of all Olympic athletes in 1994 were found to have previously suffered one or more serious skiing injuries [9]. The prevalence of ACL re-injury (same knee) has been reported to be as high as 19 % [20], and the risk of sustaining a re-injury or an additional injury was found to be significantly higher the earlier in a sports career the first injury occurred [21]; however, there is no statistical evidence that proves the risk of re-injury is higher for a pre-injured knee than for a healthy knee [39].

Athlete-related risk factors that were suggested based on expert stakeholder perceptions, as well as association with individual injury cases or injury-related variables (i.e. an indirect relation to injury risk) were ‘fatigue within a course or training session’ [25, 34, 35], ‘inappropriate tactical choices’ [26, 34, 35], ‘insufficient physical fitness’ [25, 34, 35], and ‘technical mistakes’ [26, 34, 35]. For instance, an indirect indication that fatigue and general physical fitness play an important role in injury causation might be found in the observation that most injuries occurred in the last quarter of the race [25] when athletes’ fatigue arguably becomes evident; however, a direct relation between fatigue and injury risk still needs to be verified because the higher injury rate towards the end of the race could also be explained by the increased risk-taking behavior of athletes.

Only four athlete-related risk factors have been identified with statistical evidence (i.e. a direct relation to injury risk has been proven): ‘insufficient core strength/core strength imbalance’ [24], ‘female/male sex’ [10, 15, 16, 22, 24]. ‘high skill level’ [20], and ‘unfavorable genetic predisposition’ [40]. With respect to the first, Raschner et al. [24] found an increased ACL injury risk for junior athletes with decreased core strength or core strength imbalance. With regard to the influence of sex, two studies related to WC alpine ski racing revealed that males were at higher risk for injuries in general (and for time-loss injuries in particular) than females [15, 16]. With respect to knee and ACL injuries, these and other studies found no significant sex differences [15, 16, 20, 21]; however, some studies reported females to be at higher risk [10, 22, 24]. Thus, the influence of sex might depend on the type of injury. With regard to skill level, athletes ranking in the Top 30 of the FIS world ranking list were found to have a higher risk for ACL injuries than lower ranking athletes [20], while the success of returning to sport was reported to be lower for athletes with higher career age at the time of injury [41]. Finally, a recent study reported a significant correlation between the ACL injury risk of competitive alpine skiers and their parents, and provided evidence that genetic predisposition might play an important role in injury causation [40], which is in line with expert stakeholder beliefs [34, 35].

#### Equipment-Related Injury Risk Factors

According to the perceptions of expert stakeholders, the ‘ski-plate-binding-boot’ system is a key injury risk factor as the equipment used at the time of the survey was ‘too aggressive in the ski–snow interaction’, ‘too direct in force transmission’, and ‘hard to get off the edge once the ski is carving’ [34, 35]. On the one hand, such equipment allows the skier to carve tight turns with a minimum of skidding [42–46], while, on the other hand, it might make it difficult to predict the equipment behavior and to handle the equipment once it gets out of control [43]. Driving factors for these equipment handling problems were suggested to be ‘heavy equipment weight’, ‘high standing height due to the ski-plate-binding-boot unit’, ‘skis with high torsional stiffness/homogenous bending lines’, and ‘stiff ski boots’ [34, 35]. Moreover, based on expert stakeholder perceptions and association with individual injury cases and statistical evidence, particularly the combination of ‘highly shaped’ (i.e. skis with small sidecut radii), ‘short’ and ‘wide’ skis, can be considered a major cause for increased risk of (knee) injuries in alpine ski racing [28, 34, 35, 47].

Another important equipment-related risk factor might be found in the construction characteristics of gate panels and poles, since direct gate contact has been reported to be associated with approximately 30 % of all injury cases [25] and ‘gates with high resistance’ are expected to increase the risk of hooking in [34, 35]. In addition, non-releases or inadvertent releases of bindings are frequently attributed to ACL injury mechanisms [27]. Current standard binding concepts are claimed not to be able to release adequately in all injury situations [34] as they only have limited degrees of freedom and are limited in distinguishing between the loads occurring during normal skiing and within injury situations. This might explain the expert stakeholders’ experiences that athletes typically risk a non-release of the binding rather than an inadvertent release [35]. Finally, insufficient body protection, particularly insufficient head protection, has been suggested to be an equipment-related risk factor [34, 35]. With regard to the latter, recent studies demonstrated the high frequencies and extreme loading conditions that are related to impacts of the head on the snow surface [25, 48].

#### Course-Related Injury Risk Factors

The one and only course-related risk factor in this review that was reported based solely on expert stakeholders’ beliefs was ‘high skiing speed combined with terrain transitions’ [34, 35]. From a theoretical perspective, it is plausible that if turns are set close to concave/convex terrain transitions (i.e. ‘compressions’), skiers are additionally challenged [49, 50].

All other course-related risk factors were described in the literature based on both expert stakeholder perceptions and associations with individual injury cases or injury-related variables. With regard to ‘high skiing speed combined with small turn radii’, such a combination is known to increase the acting ground reaction force [51]. In giant slalom, a recent study by Gilgien et al. [17] reported injuries to be most likely associated with high loads while turning, which is in line with the views of WC expert stakeholders [34, 35]. A similar accordance of expert stakeholder perception and indirect association with injury risk can be observed for ‘high skiing speed in general’ [15, 17, 34, 35]. In fall or crash situations, the magnitude of speed is of particular importance since speed determines the kinetic energy that has to be dissipated during a crash impact [17]. Moreover, it is plausible that increased speed reduces the time that skiers have to anticipate and adapt to technically demanding sections (e.g. jumps, rough terrain or turns) and therefore might make the incidence of mistakes more likely [17].

Generally, expert stakeholders consider jumps to be related to injuries [34, 35]. Systematic video analyses, as well as biomechanical field and simulation studies indicated an association between jumps and real injury cases and injury-related variables, respectively [17, 25, 27, 52, 53]. With respect to landing kinematics, it is known that increased overall backward lean, particularly a backward orientated trunk position, is a crucial factors for ACL loading [52]. With regard to the driving risk factor ‘inappropriate jump construction’, limited preparation time, high take-off speeds, steep take-off angles, and landings in the flat terrain can theoretically be considered to be the most dangerous characteristics of jumps [53]; however, to date no study has assessed whether there is a direct relation between jumps and injury risk.

With regard to environmental conditions and organizational safety precautions, ‘inappropriate net positions’, ‘limited spill zones’, and ‘poor visibility’ have been suggested to be dominant factors contributing to injury [34, 35], and have been found to be associated with real injury cases [25, 26]. In this connection, the positioning of B-nets in front of A-nets was perceived to increase injury risk, particularly when spill zones were small [35]. Poor visibility was mainly reported to be related to flat light, poor additional blue coloring of the snow surface, and fog [26].

#### Snow-Related Injury Risk Factors

Based on expert stakeholder perception and association with individual injury cases to date, four different snow-related risk factors have been reported in literature: ‘aggressive snow conditions’, ‘changing snow conditions’, ‘too bumpy/too smooth snow surface’, and ‘water-injected/non-injected snow’ [26, 34, 35]. With regard to ‘aggressive snow conditions’, snow temperature, snow density, and snow microstructure are known to be factors that determine the response of the snow surface to applied loads [54]. In this context, particularly cold temperatures, low humidity and artificial snow production have been suggested to be associated with aggressive snow conditions (i.e. equipment reacts immediately and loads are transmitted directly) [35]. The fundamental phenomenon of artificial snow is the small snow grain size, high snow density, and the strong bonding between neighboring snow grains (microstructure) [55], resulting in high penetration resistance and an aggressive ski–snow interaction [56]. Furthermore, ‘changing snow conditions’ within the same run might expose athletes to additional risk since alterations in the snow surface challenge the athletes in adapting their technique and setting up their equipment adequately [26, 34, 35]. With regard to the charateristics of the snow surface, contradictory views exist on whether a bumpy preparation increases or decreases injury risk. A study by Bere et al. [26] reported small bumps to be main contributors to slip-catch ACL injury mechanisms, while some of the expert stakeholders interviewed by Spörri et al. [35] argued that bumpy conditions would decrease injury risk. Different expert stakeholder perceptions also exist with regard to the use of water to prepare ski-racing slopes. While some experts argued that water injection is the preparation technique that results in the safest snow conditions because, on icy surfaces, equipment is not as reactive, others argued that, particularly at lower levels of female alpine ski racing, these conditions are risky because they bring athletes close to their physical and technical limits [35]. In fact, association between icy, water-injected slopes and individual cases of real injury situations (i.e. slip-catch cases) has been demonstrated [26].

### Potential Injury Prevention Measures

An overview of all etiology-derived potential prevention measures previously reported in the literature is presented in Tables 2, 3, 4 and 5 (right-hand side). Due to space restrictions, measures that are based on authors’ suggestions or expert stakeholder perceptions only, are not reproduced in the following sections but are presented in the aforementioned tables.

#### Athlete-Related Injury Prevention Measures

With regard to the risk factor ‘adverse crash behavior’, awareness training of how injuries occur (e.g. by explaining typical injury mechanisms) and how these can be avoided has been demonstrated to reduce serious knee sprains by up to 62 % in trained patrollers and instructors [57]. Even if these kinds of interventions might be more challenging to implement when working with competitive athletes, they could be effective for some injury situations (e.g. when the ski abruptly catches the snow surface while the skier is trying to get up after slipping out sideways; in this case, teaching athletes not to get up while they are in motion might help to prevent the occurrence of typical ACL injury mechanisms).

With regard to ‘low peripheral body temperature’, the International Olympic Committee (IOC) and the FIS follow the strategy of avoiding competitions when the effective windchill temperatures are colder than −27 °C [36]. Expert stakeholders have suggested the compulsory use of thicker racing suits with enhanced thermal insulation [35] since clothing represents the most important modifiable factor influencing injury risk when being exposed to cold temperatures [37].

With regard to the risk factor ‘pre-injury’, meaningful screening methods identifying athletes at high risk of (re)injury [58–60] might help to develop sophisticated and individualized prevention and/or return-to-sport training programs [61], and are therefore essential tools for controlling the risk of (re)injury and safely returning to sport. Guided by the current body of knowledge on non-contact ACL injury mechanisms in team sports [62], Hewett et al. [58] introduced a biomechanical screening method that assessed neuromuscular control and valgus loading during jump landings aimed at predicting the risk of prospective ACL injuries. In fact, athletes who later sustained an ACL injury showed higher knee valgus angles at the initial screening than those who remained uninjured. Since typical ACL injury mechanisms in alpine ski racing include similar loading patterns to those identified in team sports [27], the proposed jump-landing screening test might also be effective for predicting the risk of ACL injuries in competitive alpine skiers. However, as ACL injuries in alpine ski racing mostly occur in situations with an asymmetric loading distribution between the outside and inside leg (i.e. while turning) [25], and since there is only moderate correlation between knee valgus angles in drop jumps and sidestep cutting maneuvers [63], sidestep cutting-based methods might be more meaningful screening tools.

Another screening method widely discussed in the context of injury prevention in alpine ski racing is the hamstrings to quadriceps (H/Q) ratio [64, 65]. The basic idea behind this approach is that strong hamstring muscles could prevent the anterior shift of the tibia relative to the femur during typical mechanisms, leading to ACL injuries. Despite several attempts, a significant effect of optimized H/Q ratio on the ACL injury risk of competitive alpine skiers has not been demonstrated. The only difference between ACL-injured and non-injured athletes reported in literature was related to the knee joint angles at which peak hamstring torques were developed (i.e. at deeper flexion angles in non-injured athletes) [65]. A major drawback of reporting peak-to-peak H/Q ratio (i.e. the most commonly used screening approach) is that this ratio provides little information about the interaction between the two muscles in the range of motion in which ACL injuries typically occur (i.e. in deep flexion) [65]. Moreover, considering the very short period of time during which ACL injuries occur (<60 ms) [28], it is not only a question of the strength of the hamstrings and quadriceps but also a question of how rapidly these muscles can be coactivated. In view of these aspects, an alternative ‘rapid H/Q strength’ screening protocol introduced recently [59] might open new possibilities for detecting strength deficits in ACL-reconstructed athletes and the prevention of ACL injuries in general. The protocol explicitly suggests the assessment of rapid H/Q strength at joint flexion angles meaningful for alpine ski-racing injuries (70°) [59]. In addition to this alternative screening protocol, a systematic evaluation of functional lower limb asymmetry by means of phase-specific kinetic impulse during countermovement and squat jump tasks might help to better monitor the progress in rehabilitation following ACL reconstruction, and to establish objective standards for a safe return to sport [60].

With regard to ‘fatigue within a course or a training session’, an active on-hill recovery has been demonstrated to optimize blood lactate clearance and to increase run completion rates [66]. In this context, a superior physical fitness level might also be a reasonable prevention measure [25, 34, 35]. With respect to specific physical fitness factors, a recent study provided evidence suggesting that training of ‘core strength’ and avoidance of ‘core strength imbalances’ are key measures for the prevention of ACL injuries in alpine ski racing [24].

To avoid ‘technical mistakes’ while skiing, sport-specific balance or neuromuscular training might be effective prevention measures [27, 67] since wearing ski boots is known to additionally challenge the dynamic task of maintaining balance [67]. Recent studies have shown the ability of neuromuscular training programs to reduce the risk of ACL injuries in sports other than alpine ski racing [68–70].

#### Equipment-Related Injury Prevention Measures

With respect to the ‘ski-plate-binding-boot’ system, several prevention measures have been previously promoted in the literature. First, ‘reduced standing height’ is expected to reduce knee joint loading, particularly during turns with large amounts of skidding [34, 35, 43]. Moreover, reduced standing height is assumed to reduce the risk of adversely catching the ski edge [43], which is known to play a central role in the causation of ACL injuries in alpine ski racing [27]. In the downhill competition discipline, additional preventative gain of lower standing height might be found in reduced kinetic energy; however, this might only be the case if this reduction is combined with other ski geometry-related prevention measures [71].

Second, ‘skis with reduced torsional stiffness’ are perceived to be easier to get off the edge once the ski is carving and corrections are required [34]. Consequently, altering the skis’ stiffness has been suggested to increase the athletes’ safety [35]. From a theoretical perspective, it is plausible that a ski that is less stiff in torsion will less aggressively engage the snow when being edged, and will be easier to pivot or make slip, if necessary [46]. In fact, a model-based parameter study found that reduced (torsional) ski stiffness resulted in more pronounced skidding the more speed increased within a sequence of ski turns [72].

Third, less-stiff boots might help protect athletes from injury because they are less direct in force transmission and are therefore less aggressive at ski–snow interaction [34, 35], two crucial factors in the causation of skiing-related ACL injuries [28]. However, this might also compromise the athletes’ performance, and it appears to be difficult to simultaneously address both safety and performance interests. With regard to the design of ski boots, two promising approaches have been introduced. One approach is a ski boot that allows the rear spoiler to be released when posterior-directed force is applied [73]. Another approach to prevent the knee from adverse loading patterns might be found in optimized boot setups that avoid valgus misalignments [74]. For a more detailed overview of recent advances in the design and production of ski boots, the reader is referred to a recent review by Colonna et al. [75].

Fourth, it has been suggested that less-shaped and longer skis with a reduced profile width protect the health of athletes, particularly when these characteristics are combined [6, 28, 34, 35, 51, 71, 76–78]. Less-shaped skis (i.e. skis with greater sidecut radii) were found to be associated with a reduced self-steering effect (i.e. the ski turns by itself if it is edged and loaded) and less aggressive ski–snow interaction [51, 76]. These two factors are known to play a central role for the causation of ACL injuries in alpine ski racing [28]. Furthermore, less-shaped skis were found to be associated with reduced kinetic energy and lower ground reaction forces during the turn phases in which most of the injuries are known to occur [76, 77, 79]. This is in line with theoretical expectations [44, 80]. Longer skis are perceived to be safer due to increased comfort and enhanced predictability at high speeds [34, 35]. Skis with reduced profile width are expected to be less difficult for the skier to get off the edge once they are carving and corrections are needed [34]. Moreover, skis with reduced profile widths are less likely to cause the knee joint to move unfavorably close to the range of motion end positions in transversal and frontal planes, potentially decreasing the risk of degenerative knee injuries [81].

With regard to gates, the development of alternative panels/poles with less resistance or an optimized release mechanism when hooking in has been suggested by WC expert stakeholders [35]. Although such systems have become standard at FIS WC races in recent years, there is still potential for further advancements [25].

A strategy with great potential for reducing the risk of injury would be the avoidance of non-releases or inadvertent releases of bindings. However, based on what is known to date, it will be a very challenging task to design a binding system that can differentiate between adverse internal rotation and valgus loading in injury situations, and the loading patterns in normal (non-injury) skiing situations in alpine ski racing [27]. Moreover, today’s release bindings are not able to sufficiently protect the knee since their degrees of freedom are limited and only sense those forces that are translated at the boot–ski interface (i.e. forces near the ankle) [65]. Obviously, sensing additional information (e.g. a combination of upright/lateral forces at the toe and heel, strain on the back of the ski boot or injury-relevant body positions) would be needed to allow more ‘educated decisions’ as to whether the binding should release [65]. In this context, current research efforts mainly focused on the development of mechatronic bindings [82]. Another approach might be found in an innovative binding plate with load-limiting features [83]. For a more detailed overview of the current technical possibilities, the reader is referred to a recent review by Senner et al. [82].

In order to protect the athlete’s body from injury, different protective devices have been proposed in recent years, i.e. hand/arm protectors, back protectors, knee and lower-leg protectors, knee orthoses, and airbag systems [84, 85]. Although these measures are based on plausible prevention concepts and have (commonly) been implemented in recent years, their effectiveness for decreasing the risk of injury is still unclear. Once their effectiveness has been verified, additional educational activities might be required to convince coaches and athletes to wear these protective devices [86]. With regard to head injuries, it is plausible that wearing a helmet can substantially reduce the risk of a head injury. However, in alpine ski racing where helmets have been mandatory for many years, head injuries still frequently occur [19]. Thus, future research efforts should primarily focus on developing more sophisticated helmet standards that cover the full extent of potential impact loadings [48]. Most recently, some improved helmet standards have been implemented within the FIS equipment regulations [87, 88]; however, there is still room for further improvement.

#### Course-Related Injury Prevention Measures

‘High skiing speed’, particularly when combined with terrain transitions or small turn radii, was reported to be indirectly associated with high injury risk [15, 17, 34, 35]. Based on this knowledge, reducing speed at terrain transitions, speed in turns, or speed in general are reasonable, etiology-derived prevention measures. From a mechanical perspective, speed is reduced when the skier turns more out of the direction of the fall line [89], or energy is dissipated due to ski–snow friction or air drag [90–92]. With regard to the latter, racing suits with increased drag coefficients have been suggested to increase athlete safety [35]; however, for a substantial decrease in speed, not only would the suits’ permeability need to change drastically but also the suits’ cut [93]. A prevention measure with more impact on speed might be adjustments in the course setting [34]. In this context, speed was found to be controllable by increased horizontal gate distance (i.e. the gate offset), and by shorter linear gate distance (i.e. the direct distance between gate to gate) [49, 50]; however, it has to be emphasized that only substantial course setting changes might be able to effectively slow down skiers [94]. Furthermore, controlling speed by increasing the gate offset might have two major drawbacks: (i) it may increase the risk of fatigue, and (ii) it may increase the risk of out-of-balance situations [94]. Based on these considerations, preference for course settings that locally and radically slow down skiers before terrain changes or key sections, have been promoted rather than marginally, but constantly, increasing horizontal gate distances [94]. Interestingly, steeper terrain and modifications in equipment geometry were also found to be associated with lower speed [49, 71, 77, 95]. With reference to steep terrain, it has to be pointed out that terrain is a given constraint for course setters, and that in steep terrain it is the larger gate offset that causes the lower speed. On the topic of modified equipment, the preventative gain of modified geometry should not be overestimated [77]. When compared with the considerable reductions of speed that can be achieved by course-related measures, equipment-induced speed reductions are relatively small [71, 77].

With regard to ‘inappropriate jump construction’, it has been suggested that decreased take-off speeds, flat take-off angles and steep landings increase athletes’ safety [35, 53]. Moreover, a systematic training of tactical decisions and exercise regimes to improve trunk control during jump landings were suggested as prevention measures [52].

The positioning/construction of safety nets have also been reported as contributors to injuries [25, 34, 35]. As has been recently illustrated, impact simulations might be helpful tools for finding optimal net positions in future research efforts [96]. In addition, the impact on and the impact absorption of safety nets should be further investigated and improved [97, 98].

Finally, with regard to ‘poor visibility’, flat light and poor (blue) coloring of the track corridor and jump take-off zones were predominant factors associated with individual injury cases [26]. Thus, it has been suggested that repeated (blue) coloration during the entire race improves the athletes’ safety [35].

#### Snow-Related Injury Prevention Measures

Similar to playing surfaces that are known to increase injury risk in various sports [99], in alpine ski racing snow conditions might play an important role [34, 35]. Since the skier’s equipment does not react as fast on water-injected or icy snow as on aggressive snow (i.e. force is transmitted less directly between the ski and snow), water-injected and icy snow conditions are believed to be safer [34, 35]. Consequently, some expert stakeholders suggested additional water preparation to neutralize extremely aggressive conditions [34, 35], whereas the same preparation technique should be applied from start to finish if feasible [26, 34, 35]; however, additional water preparation at lower levels of female alpine ski racing should be avoided altogether [35].

### Evaluation of Prevention Measures

Finally, it has to be emphasized that only one alpine ski-racing-specific prevention measure successfully passed through all four steps of van Mechelen’s ‘sequence of prevention’ model, and that a positive impact on injury risk was only demonstrated for this particular measure: ‘less-shaped and longer skis with reduced profile width’ [47]. Interestingly, this trend is in contrast to that observed in recreational skiing, where the introduction of highly shaped and short carving skis have decreased injury rates in recent years.

## Limitations

As discussed in Sect. 2, this is a narrative review of the current literature; however, while the authors believe the review adds valuable new perspectives on the topic, two potential limitations can be identified: (i) the risk of selection bias; and (ii) the risk of subjectivity. Figure 2 was included in recognition of these limitations and to ensure that the literature search strategy and article selection process are transparent and replicable.

## Where to Go from Here to Prevent Injuries in Alpine Ski Racing?

With regard to injury epidemiology, current statistical efforts within the FIS ISS primarily assessed injuries that occurred during the competition season at WC level [12, 15, 16, 18, 19, 47]. Only a few studies included more diverse cohorts from national ski associations (not limited to athletes at WC level) [20–23]. Thus, ongoing injury surveillance at WC level should be consolidated and expanded to include a wider spectrum of skill levels (e.g. European Cup level, FIS level, and youth level), as well as the off-season/training season. However, one should keep in mind that, for this purpose, combined efforts of scientists, the FIS, and national ski associations are indispensable.

With regard to injury etiology to date, only five risk factors with statistical evidence were identified (i.e. with a proven direct relation to injury risk): ‘insufficient core strength/core strength imbalance’ [24]; ‘female/male sex’ [10, 15, 16, 22, 24]; ‘high skill level’ [20]; ‘unfavorable genetic predisposition’ [40]; and the combination of highly shaped, short, and wide skis [47]. One explanation for this might be found in the limited statistical power of epidemiological studies when dealing with a statistically ‘small’ cohort of elite athletes. Another explanation might be the multifactorial nature of injury causes in a changing outdoor environment, which further decreases the chance of successfully establishing injury etiology by the use of pure statistical approaches. Therefore, innovative alternative study designs, such as systematic video analyses [25–27], qualitative expert stakeholder interviews [34, 35], and/or biomechanical approaches [17, 28], should be recognized as essential complementary tools for the investigation of injury causes, in addition to the traditional study designs of medical research.

With regard to prevention measures, major knowledge deficits were observed regarding the evaluation of the effectiveness of potential prevention measures (see Tables 2, 3, 4, 5, prevention measures with a status of 3 or below). To date, only the combination of less-shaped and longer skis with reduced profile width was statistically confirmed to have a positive effect on injury risk in alpine ski racing [47]. At this point, it must be emphasized that, in addition to an evaluation of the direct effect of prevention measures on injury incidence and severity, a preceding assessment of the effects on injury-related variables (i.e. risk factors) might also provide essential knowledge prior to exposing athletes to unexplored prevention approaches. Such a process has recently been passed prior to, for example, implementing the FIS new equipment rules [6, 51, 71, 76, 77]; however, because this rule change, as well as other potential prevention measures described in this article, has not reached the youth age group (athletes aged 12–15 years), more research on protecting this particular group is required.

On a final note, it has to be emphasized that despite the aforementioned knowledge deficits, many of the prevention measures presented in Tables 2, 3, 4 and 5 are theoretically plausible and should therefore be proactively communicated and systematically implemented by alpine sport federations and sport practitioners, as long as no contraindications exist.
